# Randomised controlled trial of exercise training during lactation on breast milk composition in breastfeeding people with overweight/obesity: a study protocol for the MILKSHAKE trial

**DOI:** 10.1136/bmjsem-2023-001751

**Published:** 2023-10-06

**Authors:** Trine Moholdt, Emily Rose Ashby, Karina Hammer Tømmerdal, Maëliss Cynthia Chloé Lemoine, Rebecca Lyng Holm, Pål Sætrom, Ann-Charlotte Iversen, Anuradha Ravi, Melanie Rae Simpson, Guro F Giskeødegård

**Affiliations:** 1 Department of Circulation and Medical Imaging, Norwegian University of Science and Technology, Trondheim, Norway; 2 Department of Gynaecology and Obstetrics, St. Olav's Hospital, Trondheim, Norway; 3 Department of Clinical and Molecular Medicine, Norwegian University of Science and Technology, Trondheim, Norway; 4 Centre of Molecular Inflammation Research, Norwegian University of Science and Technology, Trondheim, Norway; 5 Department of Public Health and Nursing, Norwegian University of Science and Technology, Trondheim, Norway

**Keywords:** body composition, carbohydrates, exercise and/or caloric restriction effects on body weight/composition, obesity, nutrition

## Abstract

Breast milk from people with overweight/obesity may differ in composition compared with that from normal-weight people. Exercise training can modify breast milk composition in rodent models, with a beneficial impact demonstrated on the offspring’s metabolism, but whether these findings translate to humans is unclear. This trial aims to determine the effect of an exercise intervention on breast milk composition and whether an exercise-induced modification of breast milk impacts the infants’ growth and body composition. Effect of Exercise Training on Breastmilk Composition is a randomised, controlled trial with two parallel groups, one exercise group and one control group, with a 1:1 allocation. We will include a minimum of 62 exclusively breastfeeding participants, 6 weeks postpartum. The exercise intervention lasts 8 weeks and comprises 25 supervised endurance exercise sessions with moderate or high intensity. The primary outcome measure is the change in the relative concentration of the human milk oligosaccharide 3′sialyllactose in breast milk from baseline at 6 weeks postpartum to the end of the intervention period. Secondary outcomes include breast milk concentrations of other metabolites, cytokines, hormones and microRNA, maternal health outcomes, infant growth, infant gut microbiome and infant circulating microRNA. Maternal and infant outcomes will be measured before, during and after the intervention period, with a follow-up of the infants until they are 24 months old. Trial registration number NCT05488964.

**WHAT IS ALREADY KNOWN ON THIS TOPIC**

**WHAT THIS STUDY ADDS**

- This is the first randomised controlled trial to determine the effect of endurance exercise training on a comprehensive range of breast milk components.

**HOW THIS STUDY MIGHT AFFECT RESEARCH, PRACTICE OR POLICY**

- This study may provide scientific knowledge to guide the development of specific exercise recommendations for postpartum individuals with overweight/obesity.
- This study could be informative for improving infant formula by increasing knowledge of exercise’s beneficial or detrimental effects on human breast milk composition.

## Introduction

 Between 1980 and 2015, global obesity rates doubled in children aged 2–4 years and increased eightfold among 5–19 year olds.^1^ Up to 21% of the population attributable risk for childhood obesity is accounted for by maternal obesity,^2^ which implies a strong mother-to-child transmission. This transmission of obesity risk is not solely genetic. It also involves interactions between genes and an ‘obesogenic’ environment modifying gene expression (epigenetic modifications) and phenotype.^3^ During the ‘first 1000 days’ of life (from conception to the age of 2 years), humans are particularly sensitive to epigenetic modifications that may lead to childhood obesity: nutrition in this period greatly impacts later susceptibility to obesity.^4^ Breastfeeding is one factor that may be important for preventing childhood obesity, as observational data show that breastfed children have a 13% lower likelihood of becoming overweight or obese compared with formula-fed children.^5^ As such, the WHO recommends exclusive breast feeding until infants are 6 months of age to protect against obesity.^6^ However, there is evidence that breast milk composition is associated with the mother’s metabolic health.^7^,^13^

Some breast milk components, such as human milk oligosaccharides (HMOs), are associated with infant obesity, with different concentrations also observed between normal-weight lactating people and those with overweight (body mass index (BMI) 25.0–29.9 kg/m^2^) or obesity (BMI>30 kg/m^2^).^10^ Maternal obesity is further associated with other differences in the breast milk metabolome.^7 8^ Both maternal BMI and infant adiposity are associated with increased breast milk levels of the metabolites 5-methylthioadenosine, mannose, lyxitol and shikimic acid.^7 8^ The levels of breast milk components considered non-nutritive, including microRNA (miRNA) and hormones, have also been associated with maternal BMI. Breast milk is rich in miRNAs, potentially acting as epigenetic regulators.^14^ The associations between selected miRNAs in breast milk and maternal BMI have been reported, and the studies included a suggestive link to infant body composition.^11 12^ However, these two studies examined two different sets of selected miRNAs, and the evidence concerning the effect of breast milk miRNAs on infant body composition remains uncertain.

The possible role of certain breast milk components in the mother-to-child transmission of obesity is supported by several studies in mice. For instance, offspring born to lean dams and cross-fostered by obese dams displayed a dysmetabolic phenotype.^15^ In addition to maternal BMI, maternal diet, smoking, mode of delivery and gestational diabetes may affect breast milk composition.^13 16 17^ Maternal physical exercise, however, has received very little attention with respect to breast milk composition. Exercise is a substantial regulator of overall systemic metabolism and affects multiple cells, tissues and organs through the increased metabolic activity of contracting skeletal muscles. The exercise-induced health benefits stem from integrating intertissue communication through signalling molecules, hormones and cytokines collectively termed ‘exerkines’.^18^ Whether these whole-body effects of exercise also affect breast milk composition is unknown.

There is currently sparse research on the effect of exercise on human breast milk. The existing evidence comes from research undertaken before the technological advent, which now allows for detailed analyses of breast milk composition. Evidence for a protective role of ‘exercised’ breast milk on offspring obesity risk is, so far, only seen in mice.^19^ Maternal exercise was shown to increase the abundance of the HMO 3′sialyllactose (3′SL) in mouse milk, which was crucial for mediating improvements to metabolic health in the offspring.^19^ It is not known whether these findings translate to humans. Consequently, we designed a randomised controlled trial (RCT) to determine the effect of exercise during lactation on breast milk composition in people with overweight/obesity.

### Aims and hypotheses

The primary aim of the Effect of Exercise Training on Breastmilk Composition (MILKSHAKE) trial is to determine the effect of exercise during lactation on relative concentrations of 3′SL in breast milk in people with overweight/obesity. The secondary aims are to investigate if maternal exercise during lactation affects other breast milk components, maternal health outcomes, infants’ weight gain and body composition and to investigate potential mechanisms explaining the effect of exercised breast milk on infant obesity risk. Our primary hypothesis is that 8 weeks of exercise training starting 6 weeks postpartum will increase the relative concentration of 3′SL in breast milk.

## Methods

### Design and study setting

**Figure 1:**
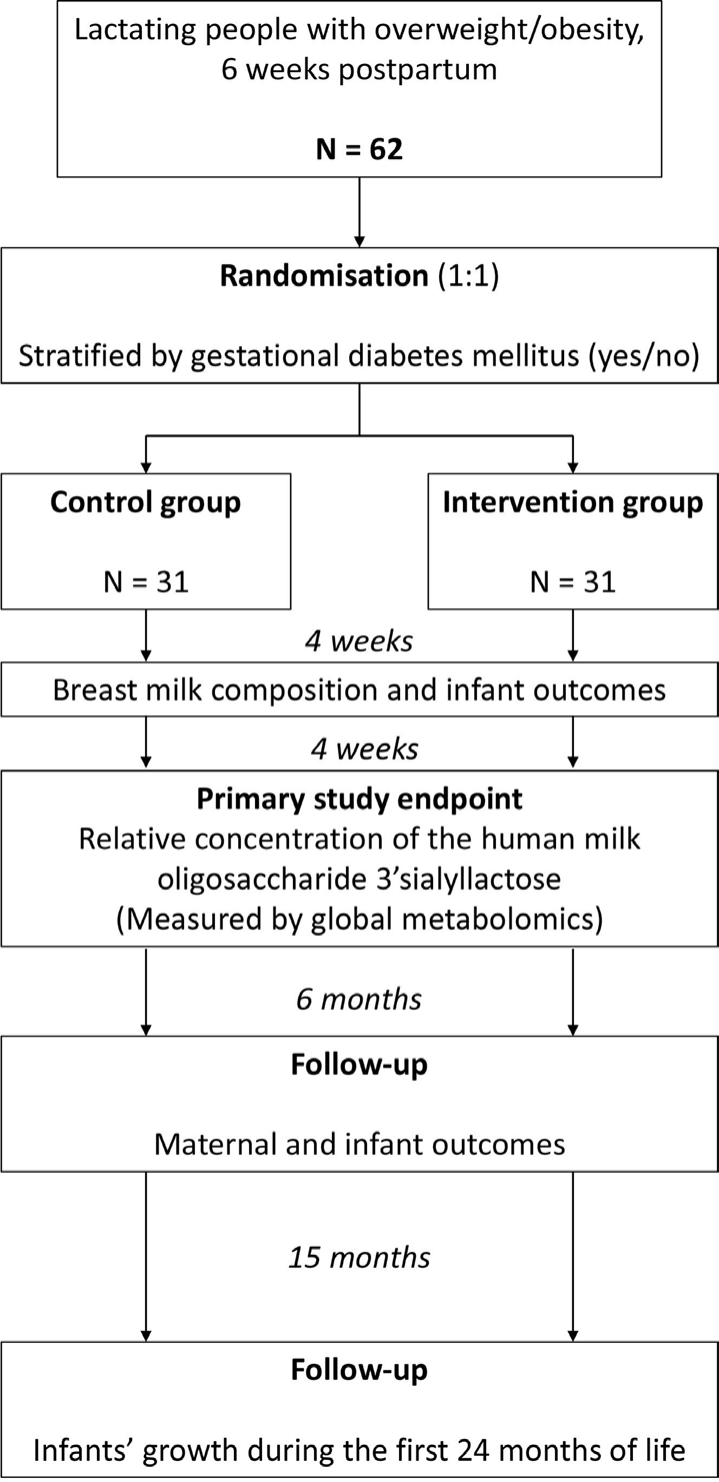
Study design. Overview of the design of the Effect of Exercise Training on Breastmilk Composition randomised controlled trial.

The MILKSHAKE trial is an RCT with two parallel groups: an intervention group (exercise training) and a standard care control group (figure 1). We will obtain data and biological material from the participants and/or their infants at baseline before randomisation (6 weeks after delivery), after 4 weeks (10 weeks after delivery), after 8 weeks (14 weeks after delivery) and when the infants are 26 weeks, 40 weeks, 12 months and 24 months. We will additionally collect growth data from local health centres. The trial is undertaken at the Norwegian University of Science and Technology (NTNU) in Trondheim, Norway, in collaboration with the St. Olav’s Hospital and Trondheim municipality. We used the Standard Protocol Items: Recommendations for Interventional Trials reporting guidelines to report the trial protocol.^20^

### Recruitment and participants

Participants will be recruited by invitations to those who have delivered a baby at the St. Olav’s Hospital maternity ward, through posts in local health centres, and via announcements on hospital and university webpages, local stores and social media. Box 1 shows the inclusion and exclusion criteria for participation.

Box 1 **Inclusion and exclusion criteria**

**Inclusion criteria**

- Age ≥ 18 years.
- Given birth to a singleton term baby (after 37 weeks +0 days).
- Prepregnancy body mass index (BMI) ≥ 25 kg/m^2^ and/or postpartum BMI ≥ 28 kg/m^2^ 1 week or more after delivery.
- Exclusively breast feeding the baby (no other foods are given) and intend to continue this practice for ≥ 8 weeks at inclusion.
- Understands oral and written Norwegian language.
- Able to attend supervised exercise sessions at the St. Olav’s Hospital.

**Exclusion criteria**

- Ongoing pregnancy.
- Known diabetes (type 1 or 2).
- Known cardiovascular diseases.
- High-intensity endurance exercise ≥ 2 times per week in the last 3 months and/or an intention to start regular endurance exercise within the next 8 weeks at baseline.

### Randomisation and allocation

Following screening and initial assessments, participants will be randomly allocated (1:1) after stratifying for gestational diabetes (yes/no) to the intervention or control groups. We will use a computer random number generator developed and administered at the Clinical Research Unit (Klinforsk), St. Olav’s Hospital, to randomly allocate participants. The allocation of participants is concealed until a participant is included. Block randomisation minimises any imbalance in group allocation, and the block sizes vary with the size of the blocks unknown to the researchers to maintain allocation concealment.

### Intervention

The exercise intervention consists of supervised endurance training, starting 6 weeks after delivery and continuing for 8 weeks. The participants will walk or run on treadmills, progressively increasing exercise volume and intensity. In the first week, they will complete two moderate-intensity sessions, lasting 40 and 45 min, respectively. After that, they will exercise three times per week for most of the remaining weeks, with four sessions in the 6th and 7th weeks (table 1). If participants miss a session in one week, they can add this session to the following week.

**Table 1:** Overview of exercise intervention

Week	Session 1	Session 2	Session 3	Session 4
1	40 min MOD	45 min MOD		
2	40 min HIIT	50 min MOD	45 min HIIT	
3	45 min HIIT	60 min MOD	45 min HIIT	
4	50 min HIIT	40 min HIIT	45 min HIIT	
5	60 min MOD	50 min HIIT	45 min HIIT	
6	60 min MOD	45 min HIIT	30 min HIIT	40 min HIIT
7	40 min HIIT	50 min MOD	50 min HIIT	60 min MOD
8	45 min HIIT	40 min HIIT	40 min HIIT	

Sessions with moderate intensity (MOD) will be continuous walking/running, and sessions with high intensity (HIIT) will be walking/running with repeated high-intensity work bouts.

The exercise intensity during the moderate-intensity sessions will be 70%–75% of the individual heart rate maximum, whereas the aim is to reach 90%–95% of the heart rate maximum during the high-intensity interval sessions. We will monitor exercise intensity by heart rate monitors (Polar, Finland). Participants needing to use ergometers other than a treadmill due to bodily pain can exercise on a stationary bike or an elliptical trainer. Participants in the control group will receive standard care and be asked to continue their habitual physical activity level. They will not be discouraged from being physically active. Neither group will receive any dietary advice.

### Experimental procedures and outcome measures

**Figure 2:**
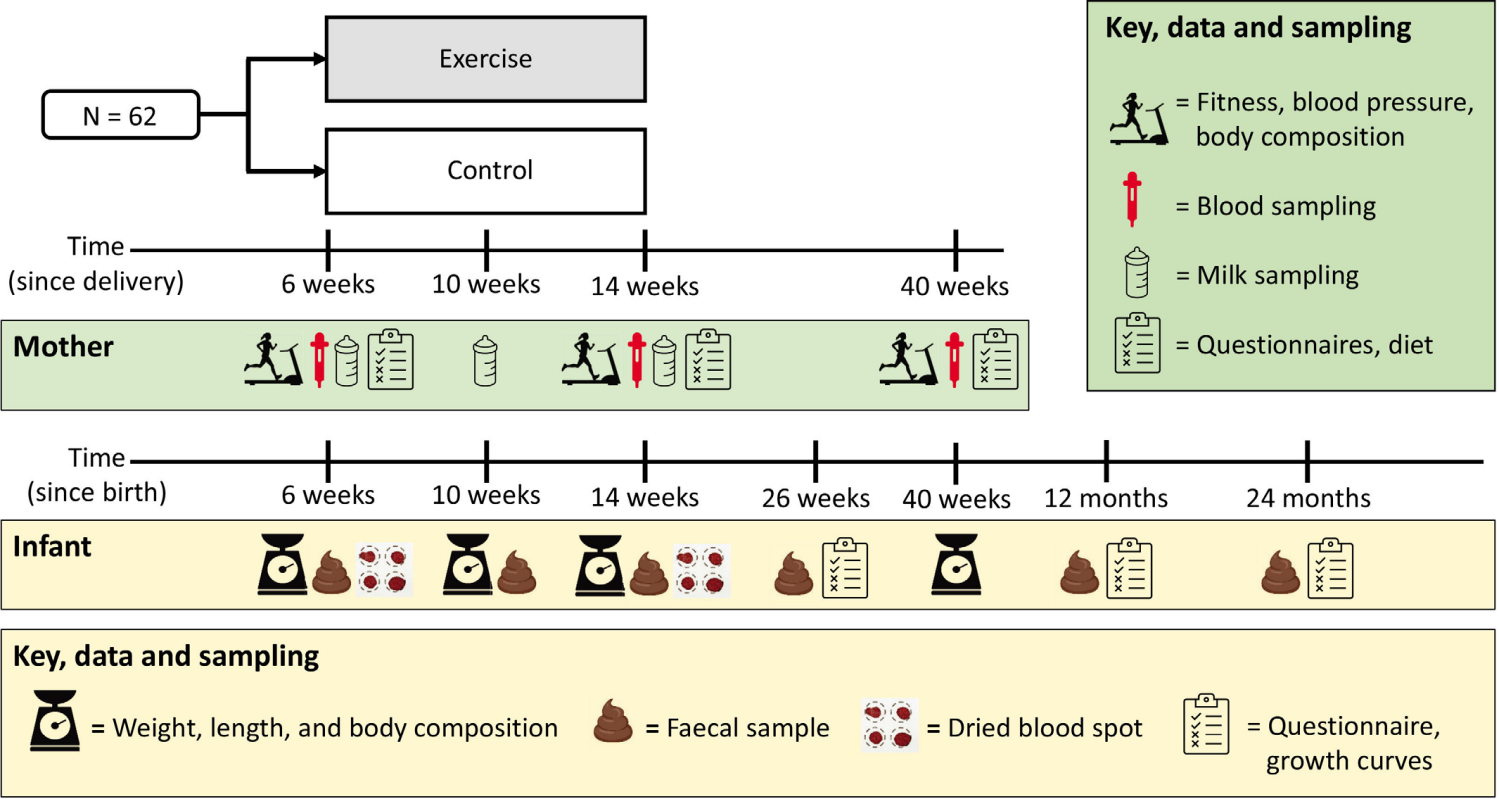
Overview of study visits schedule and procedures.

The study period spans from baseline assessments and up to the infants are 24 months old (figure 2). The participants visit the lab for assessments at baseline (6 weeks postpartum), mid-way through the intervention period (10 weeks postpartum), after the end of the 8-week intervention period (14 weeks postpartum) and after 40 weeks from delivery. We will collect data on infant growth from local health centres until the infants are 24 months old.

#### Breast milk sampling

Breast milk will be sampled at three time points: at baseline (6 weeks postpartum), after 4 weeks (10 weeks postpartum) and after 8 weeks (14 weeks postpartum). The participants will receive an electric breast pump (Medela, Switzerland). They will be given a detailed instruction sheet and a link to a video for standardised milk collection procedures. The participants will fully express from one breast to minimise differences due to hindmilk versus foremilk variations. The full sample will be gently mixed, and 40 mL will be taken for our research, with any remaining milk being left for the participants’ use. To minimise diurnal variations, the participants will express milk at the second feeding of the day (or before 09:00). They will fast for >2 hours before collection. Data collection, including breast milk and blood sampling, will be undertaken 36–48 hours after an exercise session to optimise the capture of chronic adaptations to exercise. The participants will store the breast milk in their home refrigerator until they deliver it to the laboratory the same day, transported in a container with cooling elements. Samples will be aliquoted into sterile cryotubes and frozen at −80°C until analysis. We will separate some fresh breast milk into fat, whey (skimmed) and cells by centrifugation, whereas the remaining milk will be frozen as full milk.

#### Primary outcome measure

The primary outcome measure is change in the relative concentrations of breast milk 3′SL from baseline to after 8 weeks (14 weeks postpartum). We plan to do untargeted metabolomics using ultra-high-performance liquid chromatography tandem mass spectrometry (UPLC-MS/MS; positive mode), UPLC-MS/MS (negative mode) and gas chromatography-MS at Metabolon (USA).

#### Secondary outcome measures

Secondary outcome measures include changes in concentrations of other components in breast milk, maternal health outcomes, infant growth and body composition, infant epigenetic markers (miRNA) and infant gut microbiota, as detailed below.

#### Breast milk components

In breast milk from baseline (6 weeks postpartum), 10 weeks postpartum and 14 weeks postpartum, we will determine the relative concentrations of metabolites and complex lipids using untargeted metabolomic and lipidomic profiling at Metabolon. At the same time points, we will measure breast milk concentrations of a network of 27 cytokines of relevance for the immune status (Human Cytokine Group 1 multiplex panel) with Luminex xMAP Technology on a Bio-Plex 200 system (Bio-Rad) at the Cellular and Molecular Imaging Facility at the NTNU. We will additionally measure concentrations of leptin, adiponectin, insulin, ghrelin and insulin-like growth factor-1 in breast milk at the same time points using ELISAs with a DS2 ELISA processing system (Dynex Technologies). We will profile miRNA after sample preparation for extracellular vesicle enrichment using a standard small RNA protocol and Illumina’s TruSeq SmallRNA Library Preparation Kit and sequencing with a NextSeq HighOutput flow cell on a NextSeq 500 Instrument (Illumina) at the Genomics Core Facility at NTNU.

#### Maternal outcomes

Maternal health outcome measures will be assessed at baseline, after 8 weeks (14 weeks postpartum) and after 6 months following the end of the intervention period (40 weeks postpartum) if not otherwise noted. Height will be measured only at baseline using a wall-mounted stadiometer. Body composition (total body mass, total fat mass, visceral fat mass, muscle mass and bone mass) will be estimated using bioelectrical impedance (InBody 770, Biospace CO, Korea). These measurements will be undertaken after an overnight fast, with the participants wearing light clothes and standing barefoot. BMI will be calculated as weight in kilograms divided by the square value of height in metres (kg/m^2^). Waist circumference will be measured using a metal measuring tape at the level of the belly button with the participant standing and after normal expiration.

Cardiorespiratory fitness will be measured as peak oxygen uptake during a maximum effort treadmill test using the gold standard direct expired gas analysis method (MetaLyzer II, Cortex, Germany). The test protocol will start with a 10 min warm-up and be individualised. We will report the average of the three highest consecutive 10 s measured as absolute (L/min) and relative (mL/min/kg) values. We will use the highest heart rate recorded throughout the exercise test to estimate the participant’s maximum heart rate.^21^

We will measure blood pressure with an automatic cuff (Welch Allyn, Germany) on the participants’ left arm after resting in a seated position for 15 min. We will obtain three measurements at 1 min intervals and report the average of these three measurements as diastolic and systolic blood pressure in mm Hg. We will additionally report the resting heart rate obtained by the same device.

We will sample maternal blood after an overnight fast and analyse the samples for blood lipids (total cholesterol, low-density lipoprotein cholesterol, high-density lipoprotein cholesterol, triglycerides), glucose, insulin C-peptide, high-sensitive C reactive protein and glycated haemoglobin concentrations at the St. Olav’s Hospital laboratory using their standard procedures. Additional blood will be stored for future analyses.

Participants will fill out study-specific questionnaires about their health and lifestyle, with questions on maternal background characteristics (eg, age, ethnicity and education), physical activity, medication use, gestational weight gain, infant birth weight, length, head circumference and sex. They will also complete the Psychological General Well-Being Index.^22^ We ask the participants to register their dietary intake using the FatSecret application for 4 days (out of which one is a weekend day), at baseline, in the last week of the intervention period and at 6 months after the end of the intervention period (40 weeks postpartum), and to share this information with the investigators. Participants will additionally report the type of food and supplements the infants are given at baseline, at the end of the intervention period (14 weeks postpartum), and at infant age 6 months, 12 months and 24 months. Participants who stop exclusively breast feeding during the study period will not be excluded from the study.

#### Infant outcomes

At baseline, after 4 weeks, 8 weeks and 6 months from the end of the intervention period, we will measure the infants’ weight and length and estimate their body composition. We will use a Seca 336 baby scale to measure weight and a Seca 2010 mobile measuring mat to measure length (Seca GmbH, Germany). The body composition of the infants will be estimated using bioimpedance (BioScan touch i8-nano, Maltron, UK). We will collect data from local health centres that offer surveillance of all infants in Norway, free of charge. These data will include weight, length/height and head circumference measurements when the infants are 4 and 6 weeks old and at 3, 4, 6, 8, 10, 12 and 24 months.

We will collect faecal samples from the infants at these ages: 6 weeks (baseline), 10 weeks, 14 weeks, 26 weeks, 40 weeks, 12 months and 24 months. The participants will be provided with a detailed description with photo illustrations. They will collect the samples into sterile cryoboxes before freezing them in their home freezer until transported to the laboratory. We plan to extract total faecal genomic DNA from the faecal samples using AllPrep Power Fecal DNA/RNA kits (Qiagen) and to undertake shotgun metagenomic sequencing with Illumina NovaSeq at the Genomics Facility, NTNU.

When the infants are 6 weeks (baseline) and 14 weeks (after 8 weeks), we will sample blood from their heels on a cellulose paper. These blood spots will dry completely at room temperature before storage in sterile, light-opaque plastic bags at −80°C until analysis. We will isolate total RNA from the dried blood spots and analyse for miRNA using the same miRNA sequencing described above for breast milk miRNA. We will focus, in particular, on miRNAs implicated in accelerating preadipocyte proliferation, insulin secretion by pancreatic beta-cells and glucose uptake by skeletal cells, which contribute to the pathological processes of obesity and type 2 diabetes^23^ and miRNAs linked to childhood obesity.^24^ Additional blood spots will be kept for later analyses.

#### Adherence

We will record adherence to the exercise programme as the number of sessions completed divided by the number of sessions scheduled (n = 25). To determine whether the participants have exercised with the prescribed intensity, we will calculate their average heart rate during the moderate-intensity sessions and their average heart rate during the second half of the high-intensity work bouts during the high-intensity interval sessions.

#### Sample size calculation

There is no prior research to guide the determination of clinically meaningful changes in the primary outcome measure. The sample size calculation was based on the observed effect of exercise training on the concentration of 3′SL in mouse milk, with an increase from 2500 nmol/mL (SD 200) to 2900 nmol/mL.^19^ For a two-tailed t-test to detect such a difference between groups in the level of this single HMO, with a statistical power of 0.90 and an alpha risk of 0.05, we will need five participants per group. Given the expected higher variation in humans, multivariate analyses and a predictable dropout rate of ~15%, we aim to include at least 62 women in the trial.

#### Statistical analyses

Our primary analysis will be completed according to the ‘intention-to-treat’ principle, using all obtained data irrespective of participant adherence to the intervention and completeness of outcome measures. No differences between groups are expected at baseline due to the randomised design. We will consider p value < 0.05 as statistically significant for our primary outcome measure. We will report Benjamini-Hochberg adjusted p values (q values) for the secondary outcome measures and consider q values < 0.05 statistically significant to decrease the false discovery rate due to multiple comparisons.

#### Multivariate analyses

We plan to use repeated measures analysis of variance simultaneous component analysis^25^ to determine multivariate changes in longitudinal profiling data (metabolomics, lipidomics and cytokines). Additionally, other bioinformatical analyses available during analyses will be considered. miRNA expression will be analysed by standardised bioinformatics pipelines as previously described.^26^ The limma and voom linear modelling framework^27^ will identify miRNAs with treatment-related longitudinal expression changes. Additionally, other bioinformatical analyses available during analyses will be considered. A taxonomic assignment that includes the abundance of different microbes within each microbiome from the infant microbiome will be used to calculate the alpha and beta diversity scores within and between periods. Differential abundance metrices and generalised linear mixed models (LMMs) will be used to estimate the fixed effects of alpha diversity of time on different clinical outcomes.

#### Univariate analyses

We plan to use LMMs for between-group comparisons of the maternal outcomes described and for the comparison of infant anthropometrics and body composition. Each model will include time and group×time interactions as fixed effect variables and subject (participant ID) as a random factor.^28^ In these analyses, we will consider the baseline values equal in both groups as there are no expected systematic differences between groups in RCTs. We will report estimates with 95% CIs and p values for group differences. In the case of non-normal model residuals, we will perform bootstrapping, transformations or non-parametric methods.

#### Sensitivity analyses

In the analyses of breast milk data, we will do additional analyses adjusted for the participant’s secretor status, which reflects their blood group antigens and is an important determinant of HMO abundance in breast milk. The secretor status of each participant will be determined by the high abundance (secretor) or near absence (non-secretor) of the HMO 2′fucosyllactose in the milk samples.^16^ We will also perform per-protocol analyses, in which we include only the participants in the intervention group who completed ≥ 20 exercise sessions (adherence ≥ 80%).

### Blinding

Participants and research personnel supervising the exercise training will not be blinded, as this is impossible. The personnel who measure infant growth at the local health centres will be blinded for group allocation, and we will complete baseline assessments before randomisation.

### Patient and public involvement

We have involved users in the planning of the study and will continue involving them in the implementation and dissemination. In the planning phase and after the study commencement, we got feedback on the study protocol and implementation from two nurses who are internationally certified lactation consultants. They advised us to delay the start of the intervention to at least 6 weeks postpartum since breastfeeding is often not established until this time. In communication with breastfeeding individuals, we have been made aware of the importance of offering a child-minding service for their babies while they exercise. They also advised us to schedule enough time for study visits and exercise sessions since they may have to breastfeed or change their baby’s nappy during these sessions.

### Safety monitoring and reporting

Since our exercise programme aligns with the recommendations for physical activity postpartum, we do not expect any adverse effects in the trial and have, thus, no data monitoring committee. The investigators are responsible for documentation of any adverse or serious events in the case report form and the serious adverse events report form, respectively. We will advise the participants to contact the investigators if they have any unusual symptoms. All serious adverse events will be reported to the sponsor (NTNU) within 24 hours after the investigators have been informed about the event.

### Data management and monitoring

We will enter the data required by the protocol into electronic case report forms (eCRF) using study-specific ID numbers as participant identifiers. The list that connects each participant to the data through a name list with study-specific ID numbers will be stored in a password-protected file on a secure server during the project period. We will ensure data quality by double data entry: one investigator enters the data in the eCRF and another controls these entries. The principal investigator (TM) is responsible for ensuring that data entry is complete, accurate and promptly performed. If any assessments are omitted, the reason for such omissions will be noted on the eCRFs. Corrections, with the reason for the corrections, will also be recorded. Paper copies of analyses, tests, questionnaires and logs will be stored without participants’ names, only with the study-specific ID numbers and time point of assessments. The final trial dataset will be accessible to investigators involved in the trial.

### Trial status and modifications to the protocol after trial commencement

The first participant was included on 14 November 2022. As of 11 September 2023, we have included 21 participants. Recruitment is expected to continue until the end of 2024. The follow-up 6 months after the intervention period was added in February 2023. We added maternal bone mass as a secondary outcome measure in July 2023. We changed the time for recruitment from 5–6 weeks postpartum to 6–7 weeks postpartum in August 2023.

## Discussion

The MILKSHAKE trial is, to our knowledge, the first RCT to investigate if maternal exercise training during lactation can modify breast milk concentrations of HMOs. We hypothesise that regular endurance exercise will increase the relative concentrations of 3′SL, and secondary, that the intervention will induce beneficial changes in other metabolites, cytokines, hormones and miRNA.

So far, there is limited data on the effect of maternal exercise training on breast milk composition and how specific breast milk components relate to protective effects on infant obesity risk. Lactate has been shown to increase in breast milk acutely after a maximal effort of aerobic exercise,^29^ but it is unclear if this impacts the baby. We recently reported increased breast milk concentrations of adiponectin 1 hour after high-intensity interval training.^30^ Higher breast milk concentrations of adiponectin are associated with decreased infant weight and abdominal circumference in the first year^31^; however, the effect of exercise-induced increases in breast milk adiponectin on infant growth and metabolism is unclear. Wolfs *et al* showed that higher breast milk concentrations of the brown fat-activating lipokine 12,13-diHOME were associated with reduced gain in BMI Z score from 0 to 6 months.^32^ They also reported that the concentration of this lipokine increased 90 min after moderate-intensity exercise undertaken 1 month postpartum.^32^ On the other hand, Dewey *et al*^33^ reported that regular endurance exercise did not affect total energy, lipid, protein or lactose content of breast milk. Additionally, the breast milk concentration of selected minerals and electrolytes did not change after maximal effort exercise.^34^

We will conceptualise human milk as a biological system, including comprehensive analyses of breast milk constituents and linking these data to detailed data from mothers and infants. Our goal is to be able to determine the impact of exercise-induced changes in breast milk composition on infant growth, body composition, epigenetics and gut microbiome composition to advance our understanding of the causal effect of maternal exercise during lactation on infant obesity risk. If our hypotheses are confirmed, our results will signal a conceptual change in clinical postpartum care and serve as a first example of how maternal lifestyle during lactation can modify infant obesity risk.

## References

[1] Di Cesare M, Sorić M, Bovet P (2019). The Epidemiological burden of obesity in childhood: a worldwide epidemic requiring urgent action. BMC Med.

[2] Voerman E, Santos S, Patro Golab B (2019). Maternal body mass index, gestational weight gain, and the risk of overweight and obesity across childhood: an individual participant data meta-analysis. PLoS Med.

[3] Isganaitis E (2019). Developmental programming of body composition: update on evidence and mechanisms. Curr Diab Rep.

[4] Mameli C, Mazzantini S, Zuccotti GV (2016). Nutrition in the first 1000 days: the origin of childhood obesity. Int J Environ Res Public Health.

[5] Horta BL, Loret de Mola C, Victora CG (2015). Long-term consequences of Breastfeeding on cholesterol, obesity, systolic blood pressure and type 2 diabetes: a systematic review and meta-analysis. Acta Paediatr.

[6] World Health Organization (2019). Childhood obesity surveillance initiative: Breastfeeding. https://www.euro.who.int/__data/assets/pdf_file/0017/400652/COSI-Breastfeeding-FS-ENG-LowRes.pdf.

[7] Isganaitis E, Venditti S, Matthews TJ (2019). Maternal obesity and the human milk Metabolome: associations with infant body composition and postnatal weight gain. Am J Clin Nutr.

[8] Saben JL, Sims CR, Piccolo BD (2020). Maternal Adiposity alters the human milk Metabolome: associations between Nonglucose Monosaccharides and infant Adiposity. Am J Clin Nutr.

[9] Sadr Dadres G, Whitaker KM, Haapala JL (2019). Relationship of maternal weight status before, during, and after pregnancy with breast milk hormone concentrations. *Obesity (Silver Spring*).

[10] Saben JL, Sims CR, Abraham A (2021). Human milk Oligosaccharide concentrations and infant intakes are associated with maternal overweight and obesity and predict infant growth. Nutrients.

[11] Zamanillo R, Sánchez J, Serra F (2019). Breast milk supply of Microrna associated with Leptin and adiponectin is affected by maternal overweight/obesity and influences infancy BMI. Nutrients.

[12] Shah KB, Chernausek SD, Garman LD (2021). Human milk Exosomal Microrna: associations with maternal overweight/obesity and infant body composition at 1 month of life. Nutrients.

[13] Samuel TM, Zhou Q, Giuffrida F (2020). Nutritional and non-nutritional composition of human milk is modulated by maternal, infant, and methodological factors. Front Nutr.

[14] Melnik BC, Stremmel W, Weiskirchen R (2021). Exosome-derived Micrornas of human milk and their effects on infant health and development. *Biomolecules*.

[15] Oben JA, Mouralidarane A, Samuelsson A-M (2010). Maternal obesity during pregnancy and Lactation programs the development of offspring non-alcoholic fatty liver disease in mice. J Hepatol.

[16] Seferovic MD, Mohammad M, Pace RM (2020). Maternal diet alters human milk Oligosaccharide composition with implications for the milk Metagenome. Sci Rep.

[17] Samuel TM, Binia A, de Castro CA (2019). Impact of maternal characteristics on human milk Oligosaccharide composition over the first 4 months of Lactation in a cohort of healthy European mothers. Sci Rep.

[18] Chow LS, Gerszten RE, Taylor JM (2022). Exerkines in health, resilience and disease. Nat Rev Endocrinol.

[19] Harris JE, Pinckard KM, Wright KR (2020). Exercise-induced 3'-Sialyllactose in breast milk is a critical mediator to improve metabolic health and cardiac function in Mouse offspring. *Nat Metab*.

[20] Chan A-W, Tetzlaff JM, Gøtzsche PC (2013). SPIRIT 2013 explanation and elaboration: guidance for protocols of clinical trials. BMJ.

[21] Berglund IJ, Sørås SE, Relling BE (2019). The relationship between maximum heart rate in a cardiorespiratory fitness test and in a maximum heart rate test. J Sci Med Sport.

[22] Grossi E, Compare A, Michalos AC (2014). Encyclopedia of Quality of Life and Well-Being Research.

[23] Cui X, You L, Zhu L (2018). Change in circulating microRNA profile of obese children indicates future risk of adult diabetes. Metabolism.

[24] Alfano R, Robinson O, Handakas E (2022). Perspectives and challenges of epigenetic determinants of childhood obesity: A systematic review. Obes Rev.

[25] Madssen TS, Giskeødegård GF, Smilde AK (2021). Repeated measures ASCA+ for analysis of longitudinal intervention studies with multivariate outcome data. PLoS Comput Biol.

[26] Nøst TH, Skogholt AH, Urbarova I (2023). Increased levels of microRNA-320 in blood serum and plasma is associated with imminent and advanced lung cancer. Mol Oncol.

[27] Ritchie ME, Phipson B, Wu D (2015). Limma powers differential expression analyses for RNA-sequencing and Microarray studies. Nucleic Acids Res.

[28] Twisk J, Bosman L, Hoekstra T (2018). Different ways to estimate treatment effects in randomised controlled trials. Contemp Clin Trials Commun.

[29] Wallace JP, Rabin J (1991). The concentration of lactic acid in breast milk following maximal exercise. Int J Sports Med.

[30] Holmen M, Giskeødegård GF, Moholdt T (2023). High-intensity exercise increases breast milk adiponectin concentrations: a randomised cross-over study.

[31] Mohamad M, Loy SL, Lim PY (2018). Maternal serum and breast milk adiponectin: the association with infant Adiposity development. Int J Environ Res Public Health.

[32] Wolfs D, Lynes MD, Tseng Y-H (2021). Brown fat-activating Lipokine 12,13-diHOME in human milk is associated with infant Adiposity. J Clin Endocrinol Metab.

[33] Dewey KG, Lovelady CA, Nommsen-Rivers LA (1994). A randomized study of the effects of aerobic exercise by lactating women on breast-milk volume and composition. N Engl J Med.

[34] Fly AD, Uhlin KL, Wallace JP (1998). Major mineral concentrations in human milk do not change after maximal exercise testing. Am J Clin Nutr.

