# Correction: Randomised controlled trial of exercise training during lactation on breast milk composition in breastfeeding people with overweight/obesity: a study protocol for the MILKSHAKE trial

**DOI:** 10.1136/bmjsem-2023-001751corr1

**Published:** 2024-10-10

 Moholdt T, Ashby ER, Tømmerdal KH, *et al*. Randomised controlled trial of exercise training during lactation on breast milk composition in breastfeeding people with overweight/obesity: a study protocol for the MILKSHAKE trial. *BMJ Open Sport, Exercise Medicine* 2023;9:e001751. doi: 10.1136/bmjsem-2023-001751

The funding number was listed incorrectly and has now been corrected in the online HTML and PDF.

